# Synergistic Antimicrobial and Antibiofilm Activity Optimization of a *Thymus vulgaris*–*Moringa oleifera*–*Echinacea purpurea* Ternary Ethanolic Extract Blend Against *Candida albicans* and *Streptococcus mutans* Using L-Optimal Mixture Design

**DOI:** 10.3390/microorganisms14092010

**Published:** 2026-09-10

**Authors:** Khadijah A. Altammar

**Affiliations:** Department of Biology, College of Science, University of Hafr Al Batin, P.O. Box 1803, Hafr Al Batin 31991, Saudi Arabia; khadijahaa@uhb.edu.sa

**Keywords:** *Candida albicans*, *Streptococcus mutans*, ternary mixture design, antibiofilm, phytochemical profiling, antimicrobial resistance, synergistic activity

## Abstract

Antimicrobial resistance and biofilm-associated oral infections require multi-target therapeutic strategies beyond single-extract herbal testing. Using the L-optimal mixture design approach, we optimized a ternary combination of *Thymus vulgaris*, *Moringa oleifera*, and *Echinacea purpurea* ethanolic extracts against *Candida albicans* and *Streptococcus mutans*, then characterized the optimized blend’s phytochemistry, antimicrobial and antibiofilm efficacy, time–kill pharmacodynamics, antioxidant capacity, and cytotoxic safety. Through HPLC, apigenin, chlorogenic acid, resorcinol, and ferulic acid were identified as dominant constituents. The optimal blend (Run 14: *T. vulgaris* 0.331, *M. oleifera* 0.318, *E. purpurea* 0.352) achieved a fractional inhibitory concentration index of 0.50 against both organisms, maximum inhibition zones of 3.10 cm (*C. albicans*) and 4.10 cm (*S. mutans*), biofilm inhibition of up to 93.2 ± 2.1% for *C. albicans* and 78.4 ± 2.8% for *S. mutans*, and confirmed fungicidal and bactericidal activity within 48 and 24 h, respectively. DPPH scavenging ranged from 55.19 to 68.73%, and oral epithelial cells (OEC) viability exceeded 83% at 300 µg/mL. All assays included three independent biological replicates; statistical significance was defined as *p* < 0.05. These findings identify the *T. vulgaris*–*M. oleifera*–*E. purpurea* blend as a synergistic, multi-target antimicrobial candidate with a favorable preliminary safety profile, supporting further development and evaluation against clinical isolates for oral fungal and bacterial infections.

## 1. Introduction

Antimicrobial resistance (AMR) ranks among this century’s most serious threats to global public health; the World Health Organization estimates that, without intervention, infections resistant to available drugs may account for 10 million deaths each year by 2050 [1,2].

Among the most clinically significant resistant pathogens, *Candida albicans* and *Streptococcus mutans* occupy distinct yet equally important niches in human disease. *C. albicans* is the principal causative agent of oropharyngeal and systemic candidiasis, is responsible for high mortality in immunocompromised patients, and is increasingly refractory to frontline azole and echinocandin antifungals [3]. *S. mutans*, a primary etiological agent of dental caries, exploits its capacity to form acid-producing biofilms on tooth surfaces and has demonstrated rising tolerance to chlorhexidine- and fluoride-based interventions [4,5]. A shared and critical virulence attribute of both organisms is their ability to form structured biofilms, which are sessile microbial communities encased in a self-produced extracellular matrix. This ability confers 10- to 1000-fold higher resistance to conventional antimicrobials compared with planktonic cells and is widely recognized as a major contributor to chronic microbial infections.

In response to the AMR crisis, medicinal plant extracts have attracted renewed scientific interest as valuable reservoirs of structurally diverse bioactive compounds with multi-target antimicrobial mechanisms that are inherently less prone to single-point resistance mutations [6,7]. Among the most pharmacologically investigated plants, *Thymus vulgaris*, *Moringa oleifera*, and *Echinacea purpurea* stand out for their well-documented antimicrobial, antioxidant, and anti-inflammatory properties [8]. *T. vulgaris* is rich in phenolic compounds and flavonoids that contribute to its antimicrobial activity via disruption of bacterial cell wall integrity and interference with fungal ergosterol membranes [9,10]. *M. oleifera* leaf extracts are characterized by exceptionally high apigenin concentrations along with resorcinol and pyrocatechol—compounds that generate oxidative stress in microbial cells and inhibit biofilm matrix formation [11,12]. *M. oleifera* extracts have also demonstrated strong antibacterial activity against *Clostridium perfringens* isolates, supporting their potential as natural alternatives to conventional antimicrobial agents [13]. *E. purpurea* preparations are enriched in chlorogenic acid and cichoric acid derivatives and demonstrate immunomodulatory and antimicrobial activities against a wide range of pathogens [14,15]. While each of these three plants has been individually studied for antimicrobial activity, and a small number of studies have tested arbitrary pairwise or fixed-ratio combinations of related phytochemicals, no prior work has systematically explored the full ternary composition space of *T. vulgaris*, *M. oleifera*, and *E. purpurea*, using a rigorous experimental design framework to identify a synergy-optimized blend. This represents a clear gap, which this study addresses. This combination was selected over other candidate plants because each species contributes a mechanistically distinct and non-redundant mode of antimicrobial action—cell-wall/membrane disruption (*T. vulgaris*), pro-oxidant/anti-biofilm activity (*M. oleifera*), and immunomodulatory/antimicrobial action (*E. purpurea*)—thus maximizing multi-target synergy and limiting overlapping activity.

A fundamental limitation of existing herbal antimicrobial research is its reliance on single-extract testing at arbitrary concentrations, which fails to account for the interactive effects, whether additive, antagonistic, or synergistic, that emerge when multiple phytochemically complex matrices are combined [16]. Mixture design methodologies, such as the L-optimal design approach, offer statistically rigorous frameworks for exploring the full composition space of multi-component blends, identifying L-optimal component proportions, and mathematically modeling response surfaces across all possible combinations without requiring exhaustive one-variable-at-a-time experimentation [17]. To the best of our knowledge, no study has applied L-optimal mixture design to optimize a ternary combination of *T. vulgaris*, *M. oleifera*, and *E. purpurea* extracts, nor has any study comprehensively characterized such a mixture across the full antimicrobial evaluation cascade, encompassing HPLC phytochemical profiling, antioxidant activity, antibiofilm inhibition, time–kill kinetics, and mammalian cytotoxicity, in a single integrated investigation.

We hypothesized that the distinct, complementary phytochemical profiles of *T. vulgaris*, *M. oleifera*, and *E. purpurea* ethanolic extracts, spanning flavonoids, hydroxycinnamic acids, and catechol-type phenolics, would produce concentration-dependent, multi-target antimicrobial and antibiofilm activity against *C. albicans* and *S. mutans* that could be rationally maximized through mixture design optimization. The novelty of this work lies in three specific contributions: (i) the first application of L-optimal mixture design to a thyme–moringa–echinacea ternary system, generating predictive mathematical models for antimicrobial responses as a function of blend composition; (ii) the integration of HPLC-based phytochemical quantification with mechanistically grounded discussion of structure–activity relationships across all bioassay outcomes; and (iii) the comprehensive pharmacodynamic characterization of the optimized blend through antibiofilm quantification at sub-MIC concentrations, time–kill kinetics (thus confirming the cidal versus static classification), and OEC cell cytotoxicity assessment (thus establishing a mammalian safety margin). In summary, this study aimed to determine the optimal proportions of *T. vulgaris*, *M. oleifera*, and *E. purpurea* ethanolic extracts using an L-optimal mixture design and to fully characterize the resulting mixture’s phytochemical composition, antioxidant activity, antimicrobial efficacy, antibiofilm potential, time–kill pharmacodynamics, and cytotoxic safety profile against *C. albicans* and *S. mutans*.

## 2. Materials and Methods

### 2.1. Collection of Plant Material, Processing, and Ethanolic Extraction

Dry leaves of *T. vulgaris*, *M. oleifera*, and *E. purpurea* were collected from the National Research Centre (NRC), Cairo, Egypt, identified using established morphological characteristics by experts at the NRC, and ground into a fine powder. For each species, 50 g of powdered material was macerated in 200 mL of 98% ethanol (Sigma-Aldrich, Darmstadt, Germany) in sealed amber flasks at room temperature for 48 h with periodic shaking [18]. The macerate was filtered through Whatman No. 1 filter paper (Cytiva, Marlborough, MA, USA). The residue was re-extracted twice under identical conditions, and the combined filtrates were pooled before evaporation. Combined filtrates were concentrated to dryness under reduced pressure using a rotary evaporator (Büchi AG 9230, Flawil, Switzerland) at 45 °C, and dry extracts were stored at 4 °C until use. Stock solutions were prepared in DMSO at a concentration of 100 mg/mL. Working dilutions were prepared in sterile distilled water; final DMSO was ≤0.5% *v*/*v*, which was confirmed to be non-inhibitory through solvent control experiments.

### 2.2. Phytochemical Profiling of the Extracts via HPLC

Phytochemical profiling of the three ethanolic extracts was performed through reversed-phase high-performance liquid chromatography (RP-HPLC) [19] using a Dionex UltiMate system controlled by the Chromeleon software version 7.1.3 (Dionex, Thermo Fisher Scientific, Sunnyvale, CA, USA). Prior to injection, extracts were passed through a 0.22 μm membrane filter. Separation was performed over 18 min on a C18 reverse-phase column with UV detection at 260 nm (UV_VIS_3 channel, bandwidth 1 nm), using a binary gradient of mobile phase A (0.1% formic acid in water) and mobile phase B (0.1% formic acid in acetonitrile). For flavonoid analysis, samples were injected at a volume of 20 µL, targeting six compounds, namely, apigenin, diosmin, rutin, hesperidin, kaempferol, and quercetin. For phenolic acid and related phenolic derivative analysis, samples were injected at 10 µL, targeting the following ten compounds: quinic acid, gallic acid, chlorogenic acid, resorcinol, pyrocatechol, vanillic acid, p-coumaric acid, ellagic acid, ferulic acid, and cinnamic acid. Peak identification was based on retention time matching against authentic reference standards, and quantification was performed based on the external standard method using calibration curves of peak area versus known standard concentrations. All analyses were performed in triplicate, and mean values are reported.

### 2.3. Microorganisms and Culture Conditions

*Candida albicans* ATCC 10231 and *Streptococcus mutans* ATCC 25175 were obtained from the Microbiological Resources Centre (MIRCEN), Cairo, Egypt. *C. albicans* was maintained on Sabouraud Dextrose Agar (SDA; Oxoid, Basingstoke, UK) at 25 °C [3], and *S. mutans* was cultured on Brain Heart Infusion Agar (BHIA; Oxoid, Basingstoke, UK) at 37 °C under microaerophilic conditions (5% CO_2_) [20].

### 2.4. L-Optimal Mixture Design of T. vulgaris–M. oleifera–E. purpurea Extract System Against C. albicans and S. mutans

The proportional levels of each extract in the ternary combination were determined using an L-optimal mixture design [21], subject to the fundamental mixture constraint A + B + C = 1, where A refers to *T. vulgaris* extract, B to *M. oleifera* extract, and C to *E. purpurea* extract, with each component constrained to 0.000 ≤ x_i_ ≤ 0.660. The design generated 16 experimental runs (Table 1), including pure blends, binary midpoints, overall centroid, and replicated axial checkpoints (runs 2/7, 3/13, 4/12, 8/15, and 14/16 were performed in duplicate), enabling estimation of pure error and lack-of-fit assessment. The three-dimensional simplex triangle represents the complete composition space, with mixtures at the vertices and at the centroid. Antimicrobial responses were modeled as a function of blend composition using special cubic and quadratic polynomial equations of the general form Y = α_1_A + α_2_B + α_3_C + α_12_AB + α_13_AC + α_23_BC + α_123_ABC, where Y is the inhibition zone diameter (cm); α_1_, α_2_, and α_3_ are linear term coefficients; α_12_, α_13_, and α_23_ are binary interaction coefficients; and α_123_ is the ternary interaction coefficient [22]. Model selection was based on ANOVA significance, lack-of-fit assessment, and fit statistics (R^2^, adjusted R^2^, predicted R^2^, adequate precision). The optimized blend identified by the model was subsequently used in all further antimicrobial characterization assays. All trials were carried out in triplicate.

### 2.5. Antimicrobial Activity of the Ternary Mixture of T. vulgaris–M. oleifera–E. purpurea Extracts Against C. albicans and S. mutans

The antimicrobial efficacy for each run of the mixture design was evaluated using the agar well diffusion technique [23]. Suspensions of *C. albicans* (ATCC 10231) and *S. mutans* (ATCC 25175) were first standardized to a 0.5 McFarland turbidity (equivalent to 1–2 × 10^6^ and 1–2 × 10^8^ CFU/mL, respectively), and 100 µL aliquots of each were distributed uniformly across the surface of Sabouraud Dextrose Agar (SDA; Oxoid, Basingstoke, UK) and Mueller–Hinton Agar (MHA; Oxoid, Basingstoke, UK) plates with sterile cotton swabs. A sterile cork borer was then used to cut 6 mm wells into the agar under aseptic conditions. The three stock extracts (100 mg/mL each) were combined in the volumetric ratios dictated by the corresponding mixture design run (Table 1) to yield a fixed total volume, and 50 µL of each resulting blend was pipetted into a well. Inoculated plates were incubated for 48 h at 25 °C (for *C. albicans*) or 24 h at 37 °C (for *S. mutans*). DMSO was added to the negative control wells, whereas gentamicin (10 µg/mL) and fluconazole (25 µg/mL) served as positive controls against S. mutans and C. albicans, respectively; these controls were included only to verify that the assay could detect activity and were not intended for standardized breakpoint interpretation. A digital caliper (Mitutoyo, Kawasaki, Japan) was used to measure the diameter of each inhibition zone (IZD, cm), which was recorded as the response variable in the mixture design model. Each trial was conducted in triplicate.

### 2.6. Checkerboard Assay and Fractional Inhibitory Concentration Index of Ternary T. vulgaris–M. oleifera–E. purpurea Extract Mixture Against C. albicans and S. mutans

The nature of the interactions between binary and ternary combinations of the three ethanolic extracts against *C. albicans* and *S. mutans* was assessed using the checkerboard microdilution method. Sixteen different combinations were prepared, and the minimum inhibitory concentration MIC for each combination was determined by broth microdilution in 96-well microtiter plates. Briefly, standardized inocula (0.5 McFarland, ~1–2 × 10^6^ CFU/mL for *C. albicans* and 1–2 × 10^8^ CFU/mL for *S. mutans*) were added to two-fold serial dilutions of each single extract in Mueller–Hinton Broth (for *S. mutans*) or RPMI-1640 broth (for *C. albicans*), and plates were incubated at 37 °C for 24 h *(S. mutans*) or 25 °C for 48 h *(C. albicans*). The MIC values of each extract in combination, defined as the lowest concentration showing no visible growth, were compared with those of the individual extracts [24,25]. The synergistic interaction was quantified using the Fractional Inhibitory Concentration Index (FICI), calculated as follows:

FICI=FIC of extract A+FIC of extract B+FIC of extract C

where the FIC of each extract is defined as follows:

$$FIC\;of\;extract=\frac{Concentration\;of\;extract\;in\;combination}{MIC\;of\;extract\;alone}$$

Interactions were categorized according to standard thresholds: synergy was defined as an FICI value of ≤ 0.5, an additive effect as 0.5 < FICI ≤ 1, indifference as 1 < FICI ≤ 4, and antagonism as FICI > 4. Each experiment was independently repeated three times.

### 2.7. Antibiofilm Activity of Ternary T. vulgaris–M. oleifera–E. purpurea Extract Mixture Against C. albicans and S. mutans

The antibiofilm activity of the optimized ternary extract blend against *C. albicans* and *S. mutans* was assessed using the crystal violet microtiter plate method [26]. Two-fold serial dilutions of the blend from MIC to 1/16 MIC were prepared in the appropriate broth medium supplemented with 1% D-glucose and inoculated with 100 µL of standardized microbial suspensions (1 × 10^6^ CFU/mL) in sterile 96-well flat-bottomed polystyrene plates, followed by incubation at 25 °C for 48 h (*C. albicans*) or 37 °C for 24 h (*S. mutans*) under static conditions. Planktonic cells were removed by aspiration, wells were washed three times with sterile phosphate-buffered saline (PBS, pH 7.4), and adherent biofilms were fixed with methanol for 15 min, then stained with 0.1% crystal violet for 15 min at room temperature. Excess stain was removed by rinsing with distilled water, and the bound dye was solubilized with 200 µL of 33% glacial acetic acid. Biofilm biomass was quantified by measuring the absorbance at 595 nm using a microplate reader (BioTek ELx800, USA). Percentage biofilm inhibition was calculated as follows:

$$Inhibition\;\left(\%\right)=\frac{OD\;control-OD\;treated}{OD\;control}\times 100$$

where OD control is the absorbance of untreated wells and OD treated is the absorbance of extract-treated wells. All experiments were performed in triplicate. Additionally, the morphological effects of treatment at the MIC on biofilm structure and cell surface integrity were visualized via scanning electron microscopy (SEM), which was performed on both treated and untreated (control) *C. albicans* and *S. mutans* biofilms fixed and prepared according to standard SEM protocols.

### 2.8. Time–Kill Kinetics Study of Ternary T. vulgaris–M. oleifera–E. purpurea Extract Mixture Against C. albicans and S. mutans

The time–kill kinetics of the optimized ternary extract mixture were determined following the CLSI M26-A guidelines with slight modifications [27]. Standardized suspensions of *C. albicans* and *S. mutans*, prepared as described in Section 2.3 (0.5 McFarland, ~1–2 × 10^6^ and ~1–2 × 10^8^ CFU/mL, respectively), were treated with the optimized mixture at MIC, ½ MIC, and ¼ MIC, or left untreated as growth controls, in 96-well microtiter plates containing 0.5 mL of Sabouraud Dextrose Broth (*C. albicans*) or Mueller–Hinton Broth (*S. mutans*). Cultures were incubated at 25 °C (*C. albicans*) or 37 °C (*S. mutans*) with continuous orbital shaking at 150 rpm. At 0, 2, 4, 6, 8, and 24 h, 100 µL aliquots were aseptically withdrawn, serially diluted 10-fold in sterile phosphate-buffered saline (PBS, pH 7.4), and 100 µL was plated in duplicate on the appropriate solid agar medium. After incubation at their respective temperatures for 24–48 h, colonies were enumerated and the results are expressed as log_10_ CFU/mL. Fungicidal and bactericidal activity was defined as a ≥3 log_10_ reduction in viable count relative to the initial inoculum at time zero [28]. MIC values were determined via broth microdilution prior to these assays. All assays were performed in triplicate.

### 2.9. DPPH Radical Scavenging Activity

The antioxidant capacity of the three ethanolic extracts was assessed via a DPPH (2,2-diphenyl-1-picrylhydrazyl) radical scavenging assay, following methodology adapted from earlier work [29]. In brief, freshly prepared DPPH solution (0.2 µM in methanol, 100 µL) was mixed with 100 µL of extract at concentrations spanning 1.5–50 mg/100 mL, and the mixtures were left to incubate in the dark at room temperature for 30 min. Absorbance readings were then taken at 517 nm on a UV/Vis spectrophotometer. The percentage of DPPH radicals scavenged was determined using the following equation [30]:

$$DPPH\;Inhibition\;\left(\%\right)=\frac{Abs\;control-Abs\;sample}{Abs\;control}\times 100$$

where Abs control is the absorbance of the DPPH solution without extract and Abs sample is the absorbance with it. All measurements were carried out in triplicate, and the results are expressed as mean percentage DPPH radical scavenging activity (%).

### 2.10. Cytotoxicity Assay Against OEC Cells

To assess the cytotoxic safety of the optimized ternary extract blend, testing was performed on OEC cells (oral epithelial cells) sourced from Nawah Scientific (Cairo, Egypt). Cells were maintained in DMEM (Gibco, Carlsbad, CA, USA) supplemented with 10% fetal bovine serum (FBS) and antibiotics (100 U/mL penicillin, 100 µg/mL streptomycin), under standard culture conditions (37 °C, humidified, 5% CO_2_). Cells between passages 4 and 10 were plated in 96-well plates at a density of 1 × 10^4^ cells per well and given 24 h to attach. Following a PBS wash, cells were exposed for 24 h to serial two-fold dilutions of the optimized blend (concentration range: 0.03–300 µg/mL) prepared in DMEM containing 2% FBS. Negative controls consisted of untreated cells, while doxorubicin (0.1–10 µg/mL) served as the positive cytotoxic control. Cell morphology was recorded using phase-contrast microscopy (Nikon Eclipse Ti, Tokyo, Japan; 200× magnification). Viability was then quantified by adding 20 µL of MTT solution (5 mg/mL, Sigma-Aldrich) to each well and incubating for 4 h at 37 °C; the resulting formazan crystals were dissolved in 200 µL DMSO, and absorbance was read at 570 nm (with a 630 nm reference wavelength) on a microplate reader (BioTek Epoch, Agilent Technologies, Santa Clara, CA, USA). Cell viability was then calculated using the following formula [31]:

$$Cell\;viability\;\left(\%\right)=\frac{OD\;treated}{OD\;control}\times 100$$

The half-maximal cytotoxic concentration (CC_50_) was determined through non-linear regression analysis. All experiments were performed in triplicate.

### 2.11. Statistical Analysis

All experiments were conducted in triplicate with three independent biological replicates (n = 3), and data are presented as mean ± standard deviation (SD). Statistical significance was determined using one-way analysis of variance (ANOVA) followed by Tukey’s post hoc multiple comparison test; differences were considered statistically significant at *p* < 0.05 (95% confidence level). Mixture design modeling, response surface analysis, and ANOVA of the special cubic and quadratic models were performed using the Design-Expert^®^ software version 13 (Stat-Ease Inc., Minneapolis, MN, USA). All other statistical analyses were performed using the SPSS software version 26.0 (IBM Corp., Armonk, NY, USA).

## 3. Results

### 3.1. HPLC-Based Phytochemical Profiling of T. vulgaris–M. oleifera–E. purpurea Extracts

**Figure 1:**
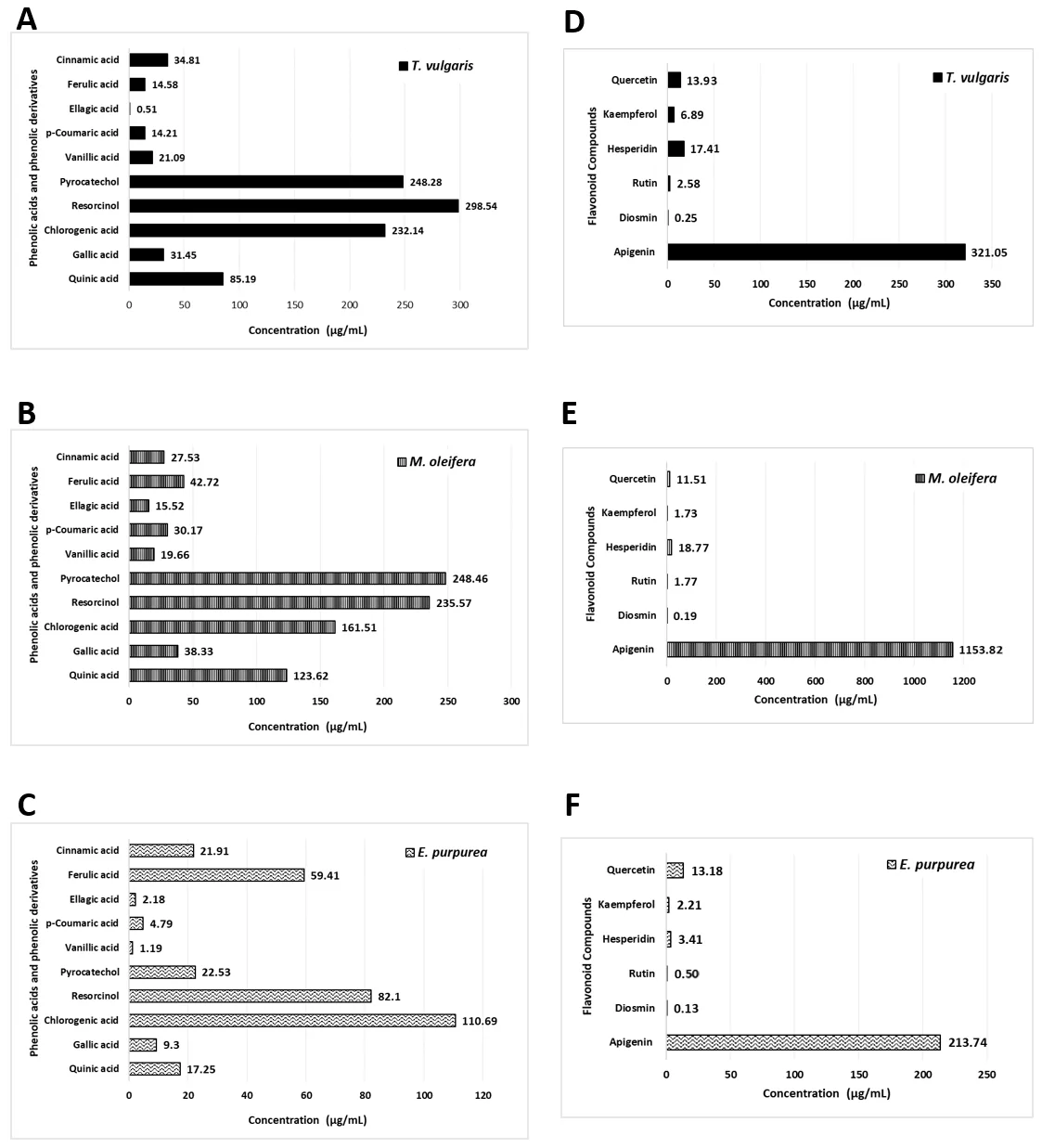
HPLC-identified Phenolic acids and derivatives and Flavonoids in the ethanolic extracts of *T. vulgaris *(**A**,**D**), *M. oleifera *(**B**,**E**), and *E. purpurea *(**C**,**F**).

Reversed-phase HPLC analysis identified ten phenolic acids and related phenolic derivatives (Figure 1)—including quinic, gallic, chlorogenic, resorcinol, pyrocatechol, vanillic, p-coumaric, ellagic, ferulic, and cinnamic acids—and six flavonoids—namely, apigenin, diosmin, rutin, hesperidin, kaempferol, and quercetin—across all three ethanolic extracts (Supplementary File Figure S1). Among the flavonoids, apigenin was dominant in *M. oleifera* (1153.82 µg/mL) and identified less frequently in *T. vulgaris* (321.05 µg/mL) and *E. purpurea* (213.74 µg/mL). Hesperidin accumulated more in *M. oleifera* (18.77 µg/mL) and *T. vulgaris* (17.41 µg/mL) relative to *E. purpurea* (3.41 µg/mL), while kaempferol was higher in *T. vulgaris* (6.89 µg/mL) relative to *M. oleifera* and *E. purpurea*. Quercetin was notably conserved across all three extracts (11.51–13.93 µg/mL). Among phenolic acids and related phenolic derivatives (Figure 1), resorcinol and pyrocatechol dominated in *T. vulgaris* (298.54 and 248.28 µg/mL) and *M. oleifera* (235.57 and 248.46 µg/mL), with markedly lower levels in *E. purpurea* (82.10 and 22.53 µg/mL). Chlorogenic acid was present in substantial amounts across all extracts (110.69–232.14 µg/mL), while ferulic acid was the defining phenolic of *E. purpurea* (59.41 µg/mL), exceeding its levels in *M. oleifera* (42.72 µg/mL) and *T. vulgaris* (14.58 µg/mL) by up to 4-fold, reflecting a notable accumulation of hydroxycinnamic acids in that extract.

### 3.2. L-Optimal Mixture Design of Ternary T. vulgaris–M. oleifera–E. purpurea Extract Mixture Against C. albicans and S. mutans

**Figure 2:**
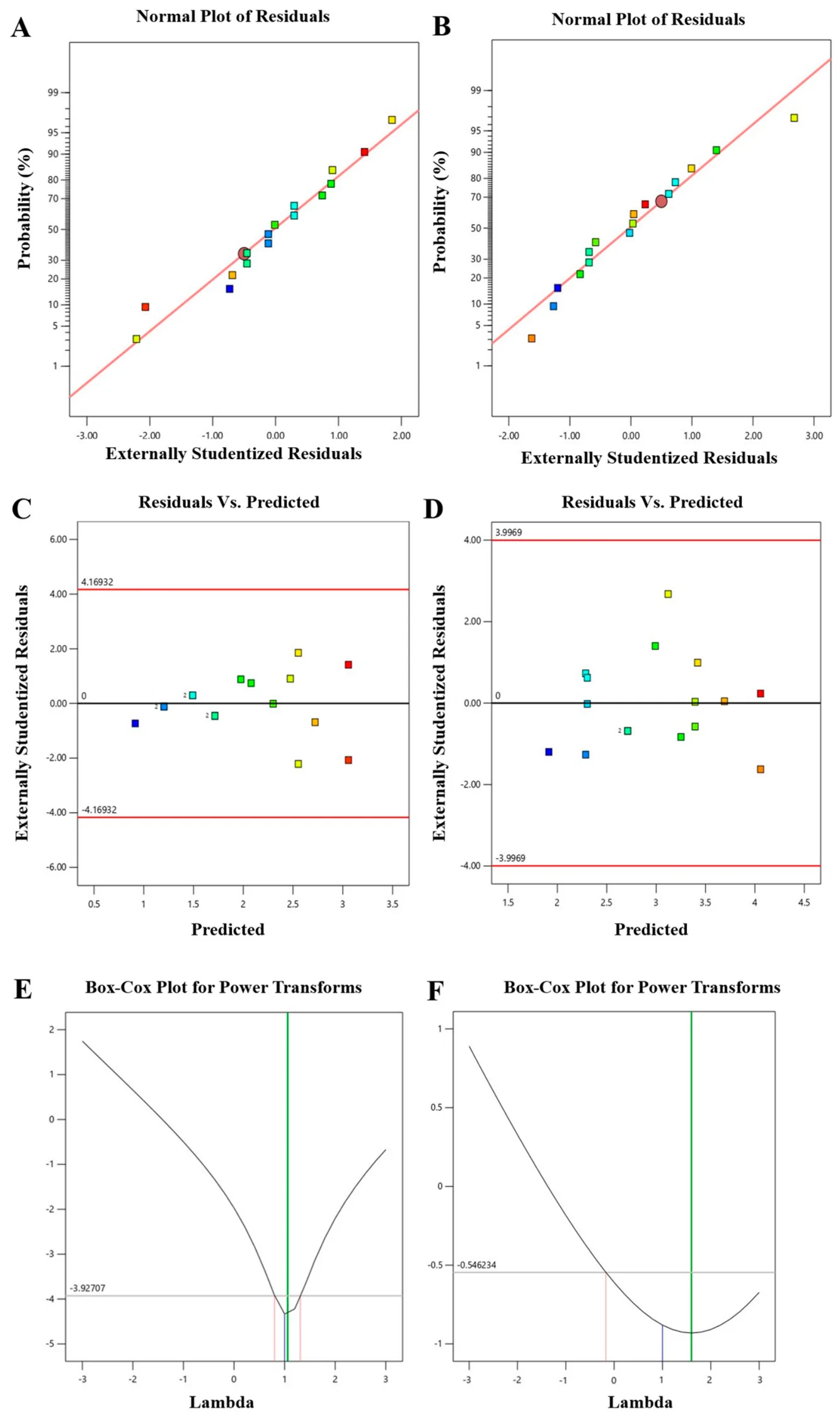

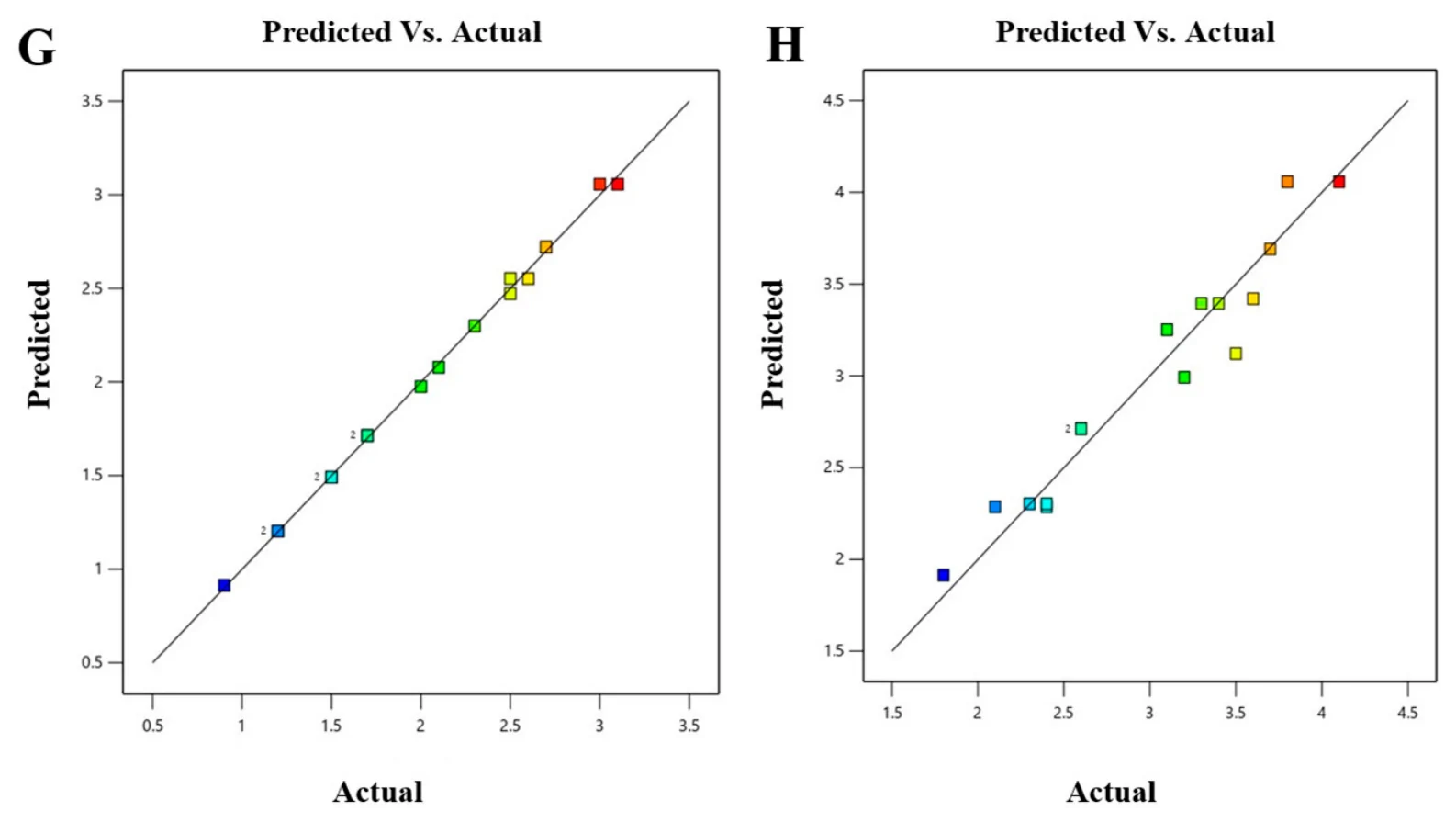
Diagnostic plots for the mixture design models of antifungal activity against *C. albicans* (left panels: (**A**,**C**,**E**,**G**)) and antibacterial activity against *S. mutans* (right panels: (**B**,**D**,**F**,**H**)). (**A**,**B**) Normal probability plots. (**C**,**D**) Residuals versus predicted plots. (**E**,**F**) Box–Cox power transformation plots. (**G**,**H**) Predicted versus actual value plots.

**Figure 3:**
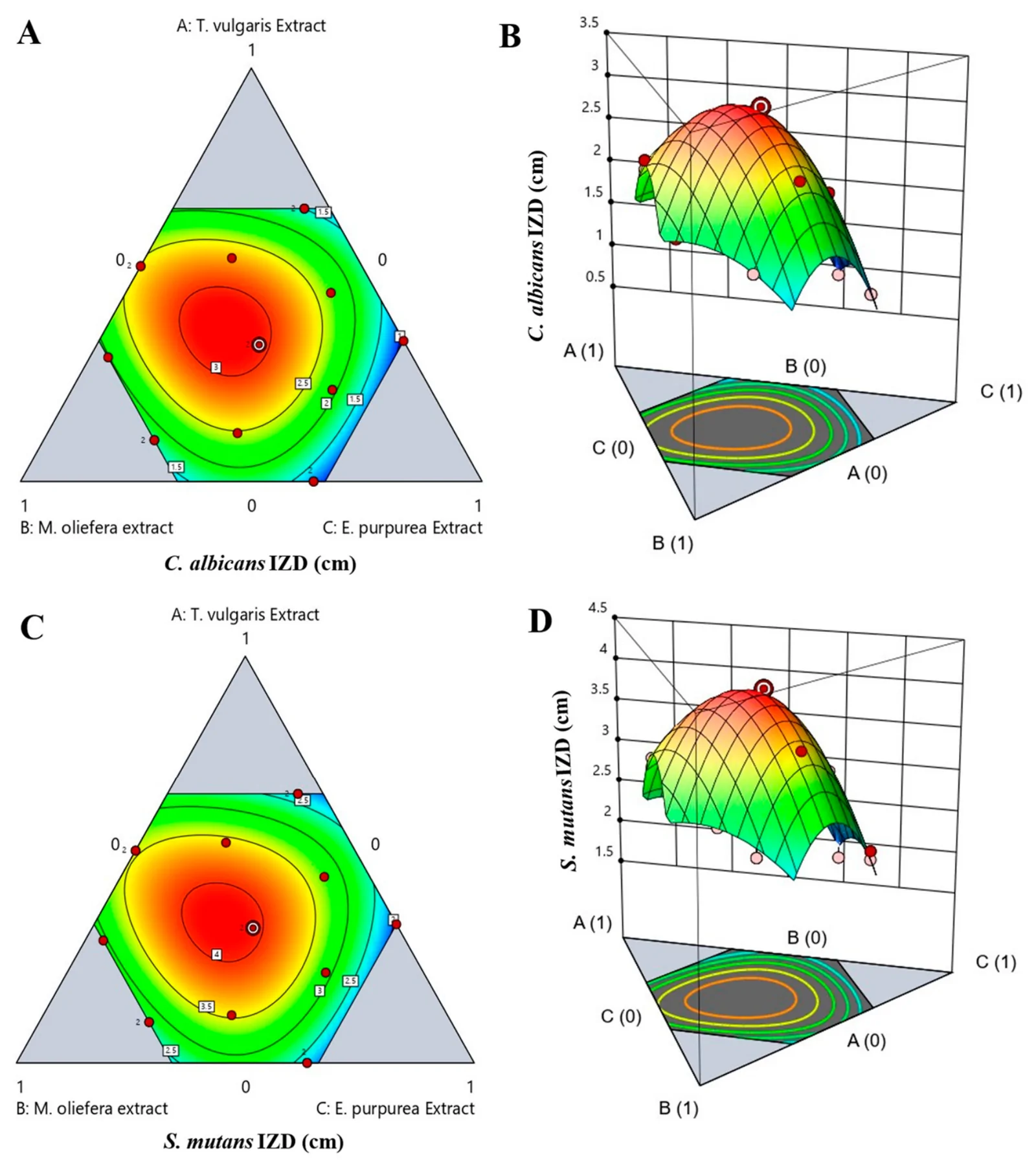
Mixture design response surface plots illustrating the effect of extract combinations on inhibition zone diameter (IZD) against *C. albicans* (**A**,**B**) and *S. mutans* (**C**,**D**). (**A**,**C**) Two-dimensional contour plots and (**B**,**D**) three-dimensional response surface plots.

The L-optimal mixture design (16 runs) presented in Table 2 was used to evaluate the combined antimicrobial activity of thyme (A), moringa (B), and echinacea (C) extracts against *C. albicans* and *S. mutans*. Inhibition zone diameter (IZD) values ranged from 0.90 to 3.10 cm and 1.80 to 4.10 cm for *C. albicans* and *S. mutans*, respectively. The mixture in Run 14 (Run 14: A = 0.331, B = 0.318, C = 0.352) produced the highest inhibition zones for both pathogens. The *C. albicans* data, shown in Table 3, are best described by a special cubic model (F = 776.69, *p* < 0.0001; R^2^ = 0.9981; CV = 1.88%; adequate precision = 84.823), while a quadratic model best fits *S. mutans* (F = 31.92, *p* < 0.0001; R^2^ = 0.9410; CV = 6.80%; adequate precision = 17.197). All binary interaction terms (AB, AC, BC) were significant (*p* < 0.0001) for both organisms, and the ternary ABC term was significant for *C. albicans* (*p* = 0.0002). The regression equations (pseudo components) were as follows: IZD (*C. albicans*) = −3.43A − 3.41B − 4.49C + 23.93AB + 22.47AC + 22.86BC − 22.41ABC and IZD (*S. mutans*) = −1.33A − 1.35B − 1.21C + 18.98AB + 14.12AC + 15.40BC. Diagnostic plots (Figure 2) confirm model adequacy. Normal probability plots (Figure 2A,B) show residuals aligning along the reference line. Residuals versus predicted plots (Figure 2C,D) depict random scatter within control limits (±4.17 for *C. albicans*; ±3.97 for *S. mutans*). Box–Cox plots (Figure 2E,F) indicate that no transformation is needed for the *C. albicans* model (λ = 1, minimum ln (ResidualSS) = −3.927), while a mild power transformation (λ = −0.546) is indicated for the *S. mutans* model. Predicted versus actual plots (Figure 2G,H) show concordance between model-predicted and observed values for both responses. Contour and response surface plots (Figure 3) show that the highest inhibition zone values for *C. albicans* (Figure 3A,B) are concentrated in a central region of the mixture space. A similar pattern is observed for *S. mutans* (Figure 3C,D), with peak antibacterial response near the interior of the simplex.

### 3.3. Checkerboard Assay and Fractional Inhibitory Concentration Index

FIC indices for all binary and ternary extract combinations against both pathogens are summarized in Table 4. Against *C. albicans*, thyme/moringa (A/B) was additive (FICI = 1.00), with the combined MIC of both thyme and moringa reduced 2-fold from their single-extract baseline (0.125 to 0.0625 mg/mL for both). Thyme/echinacea (A/C) was synergistic (FICI = 0.50), with thyme reduced 4-fold (0.125 to 0.03125 mg/mL) and echinacea reduced 4-fold (0.500 to 0.125 mg/mL). Moringa/echinacea (B/C) fell in the additive range (FICI = 0.75), with moringa reduced 4-fold (0.125 to 0.03125 mg/mL) and echinacea reduced 2-fold (0.500 to 0.250 mg/mL). The ternary combination yielded the strongest outcome (FICI = 0.50; combined MIC values of 0.015625, 0.03125, and 0.0625 mg/mL for thyme, moringa, and echinacea, respectively, reduced 8-fold, 4-fold, and 8-fold from single-extract MICs of 0.125, 0.125, and 0.500 mg/mL), which was classified as synergy. Two of the three binary combinations were classified as indifferent against *S. mutans*. Thyme/moringa (A/B) produced a FICI of 1.50 (combined MIC 0.125/0.0625 mg/mL against single MICs of 0.125 mg/mL each, with thyme unchanged), thyme/echinacea (A/C) produced a FICI of 1.00 (combined MIC 0.0625/0.250 mg/mL against single MICs of 0.125 and 0.500 mg/mL, respectively), and moringa/echinacea (B/C) produced a FICI of 1.50 (combined MIC 0.125/0.250 mg/mL against single MICs of 0.125 and 0.500 mg/mL, respectively, with moringa unchanged). The ternary combination again produced synergy (FICI = 0.50), with combined MIC values of 0.03125, 0.015625, and 0.0625 mg/mL for thyme, moringa, and echinacea, respectively, reduced 4-fold, 8-fold, and 8-fold from single-extract MICs of 0.125, 0.125, and 0.500 mg/mL.

### 3.4. Antibiofilm Activity of Ternary T. vulgaris–M. oleifera–E. purpurea Extract Mixture Against C. albicans and S. mutans

**Figure 4:**
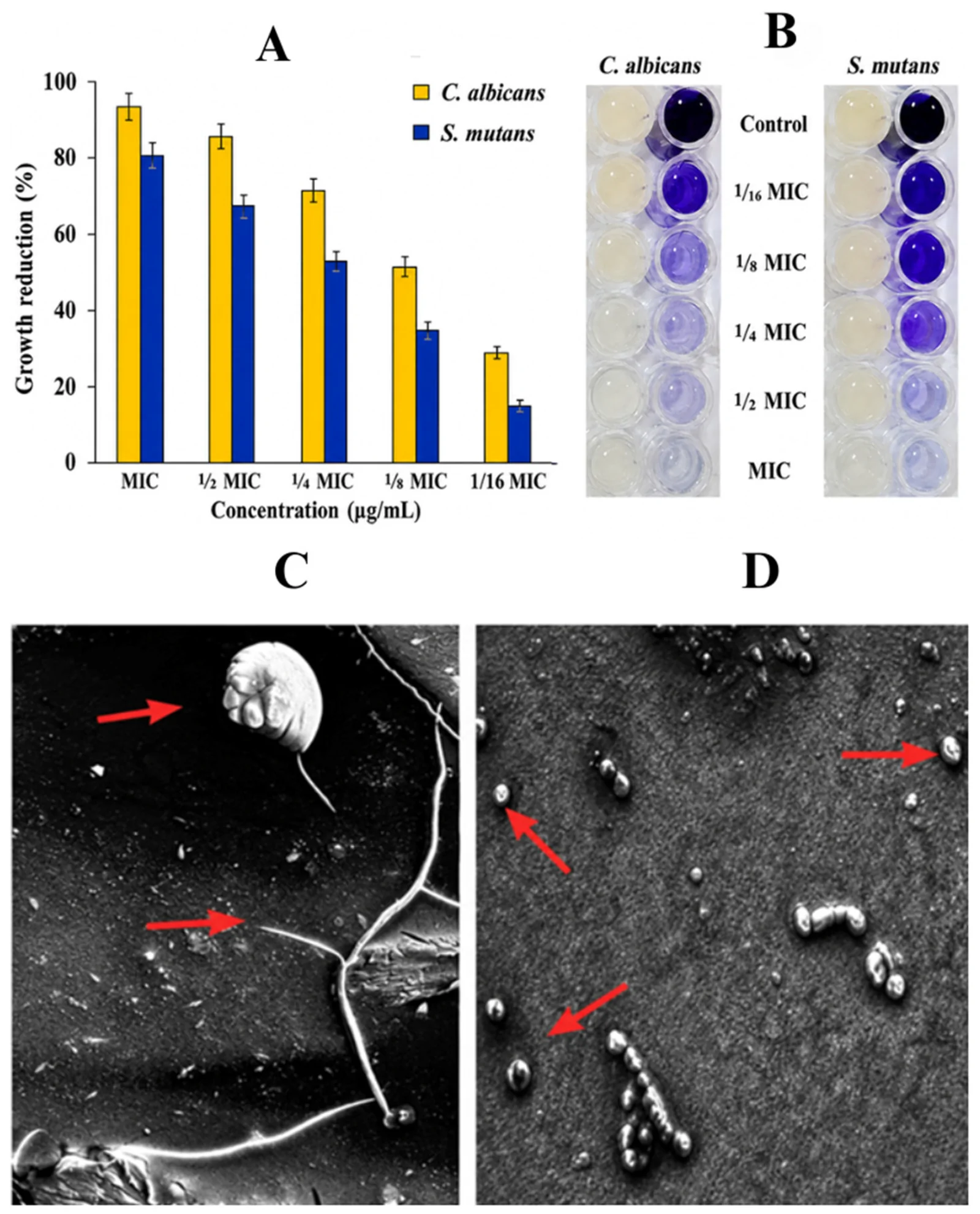
Concentration-dependent antibiofilm activity of the optimized thyme–moringa–echinacea ternary mixture against *C. albicans* and *S. mutans*. (**A**) Percentage growth reduction at serial 2-fold dilutions (MIC to 1/16 MIC). (**B**) Crystal violet-stained microtiter wells. (**C**,**D**) SEM micrographs showing the morphological effect of treatment on *C. albicans* (**C**) and *S. mutans* (**D**), with pronounced surface disruption, collapse, and deformation. Red arrows indicate damaged cells.

The ternary *T. vulgaris*–*M. oleifera*–*E. purpurea* mixture produced pronounced, concentration-dependent biofilm inhibition against both *C. albicans* and *S. mutans* across all five sub-MIC concentrations, with all inter-level comparisons statistically significant (*p* < 0.05, Tukey’s HSD; Figure 4). Inhibition ranged from 93.2 ± 2.1% to 23.6 ± 2.2% for *C. albicans* and from 78.4 ± 2.8% to 12.4 ± 2.1% for *S. mutans* between MIC and ^1^⁄_16_ MIC, with *C. albicans* consistently exceeding *S. mutans* by 11.2–14.8 percentage points across the tested concentration range.

### 3.5. Time–Kill Study of T. vulgaris–M. oleifera–E. purpurea Ternary Extract Mixture Against C. albicans and S. mutans

**Figure 5:**
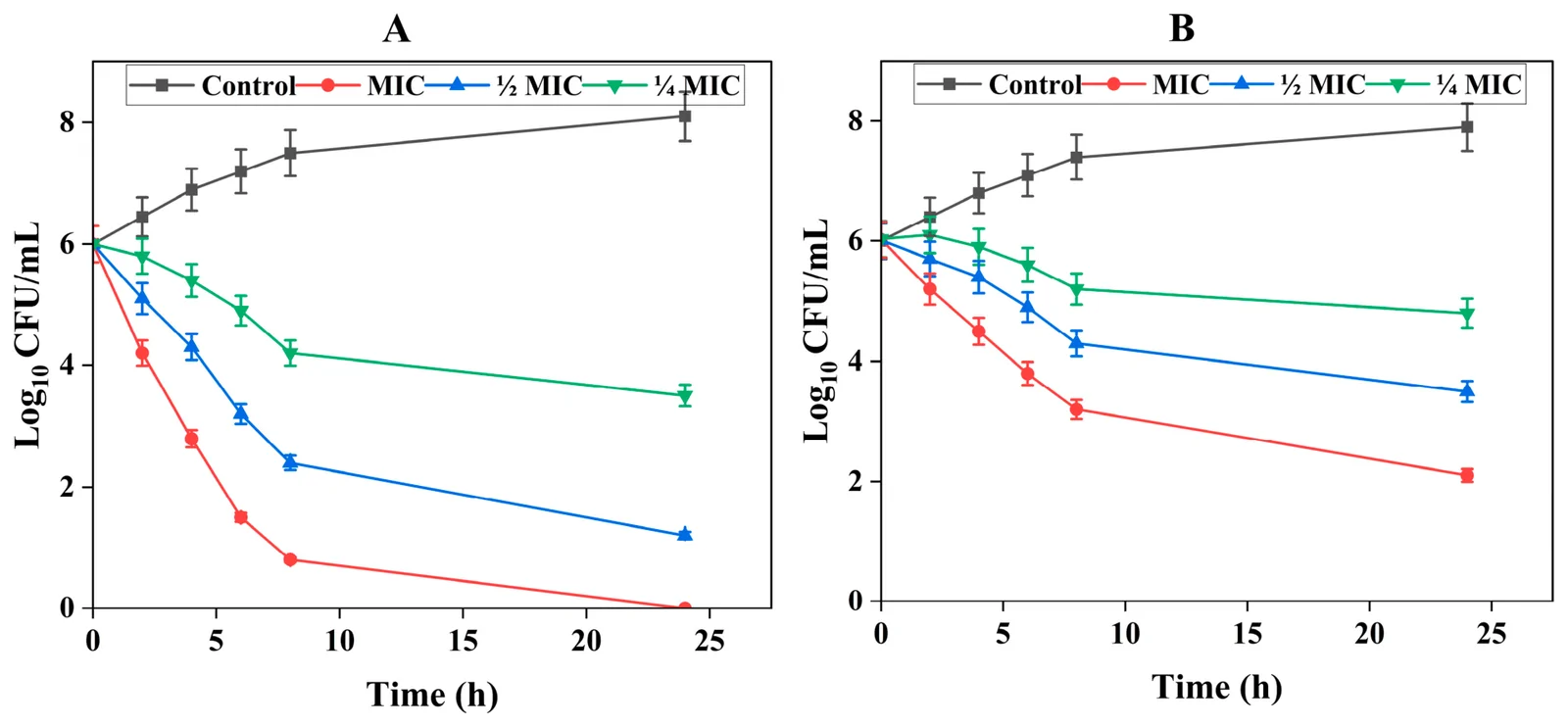
Time–kill kinetics of the optimized thyme–moringa–echinacea ternary blend against *C. albicans* (**A**) and *S. mutans* (**B**) at MIC, ½ MIC, and ¼ MIC over 24 h.

At 24 h, after starting from a standardized inoculum (6.0 log CFU/mL), untreated controls reached 8.10 ± 0.41 and 7.90 ± 0.40 log CFU/mL for *C. albicans* and *S. mutans*, respectively, while the ternary mixture exerted concentration- and time-dependent killing against both organisms (Figure 5). Against *C. albicans*, MIC treatment produced a progressive 4.5-log reduction by 6 h (6.0 to 1.5 log CFU/mL) and complete eradication by 24 h (no colonies recovered, <1 log CFU/mL/below the limit of detection), confirming fungicidal activity. Treatment at ½ MIC yielded a 4.80-log reduction by 24 h (6.0 to 1.2 log CFU/mL), while ¼ MIC produced a 2.50-log reduction (6.0 to 3.5 log CFU/mL). Against *S. mutans*, killing was slower and near-linear, achieving a 3.93-log reduction at MIC (6.03 to 2.10 log CFU/mL) by 24 h, meeting the ≥3-log bactericidal threshold. Treatments at ½ MIC and ¼ MIC produced sub-bactericidal reductions of 2.50 log CFU/mL (6.01 to 3.50) and 1.23 log CFU/mL (6.03 to 4.80), respectively, consistent with bacteriostatic activity at sub-threshold concentrations.

### 3.6. Total Antioxidant Activity (DPPH Radical Scavenging Assay)

**Figure 6:**
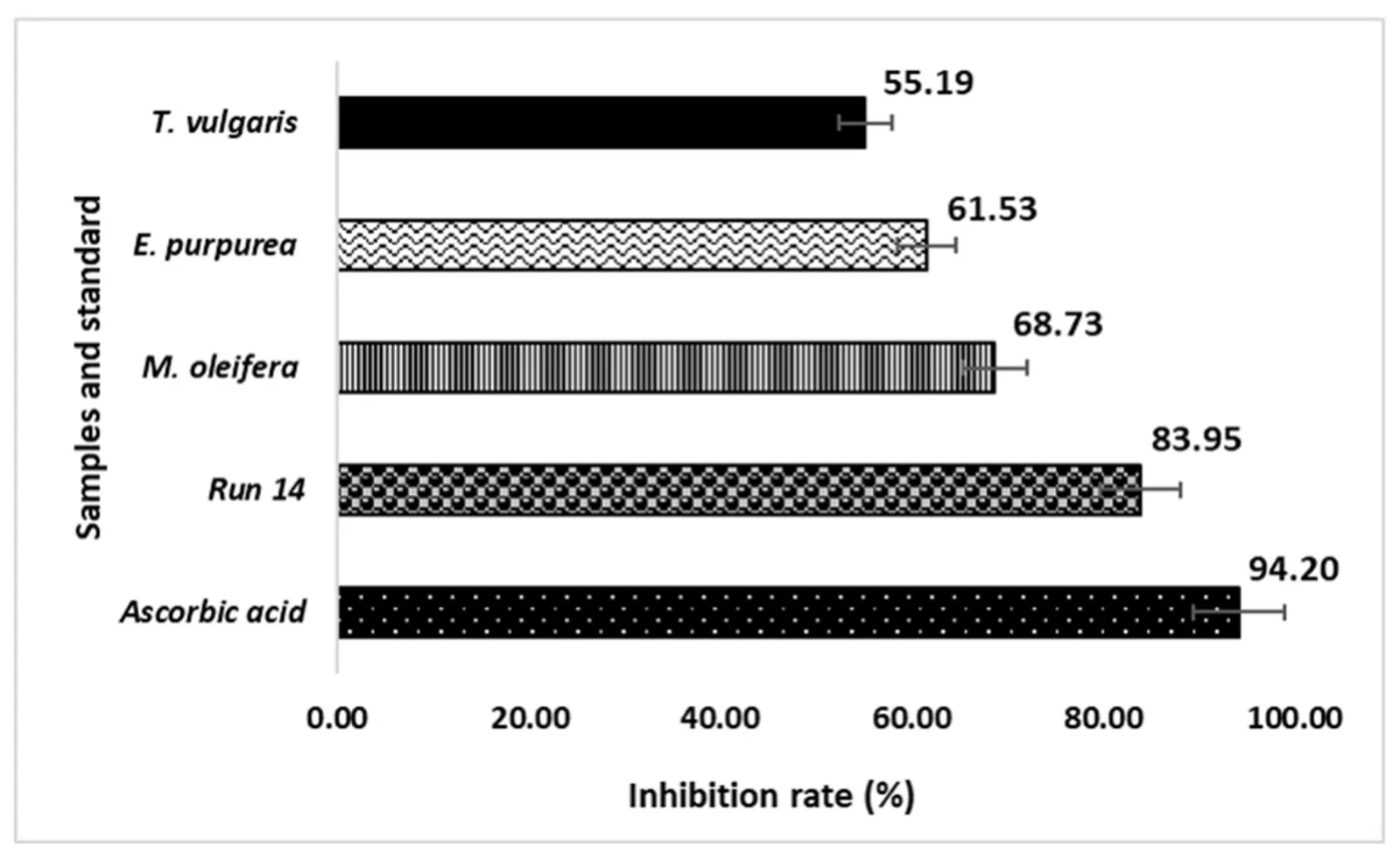
Antioxidant activities of *T. vulgaris*, *M. oleifera*, and *E. purpurea* extracts and their optimized ternary mixture (Run 14) using the 2,2-diphenyl-1-picrylhydrazyl (DPPH) free radical scavenging assay.

To examine the extracts’ antioxidant potential, they were evaluated for free radical scavenging potential using a DPPH free radical scavenging assay. All three extracts exhibited measurable DPPH radical scavenging activity (55.19–68.73%), ranking moringa > echinacea > thyme, a trend closely paralleling their total flavonoid and phenolic acid content (Figure 6). Moringa’s superior activity (68.73%) is attributable to its exceptionally high content of apigenin, pyrocatechol, resorcinol, and chlorogenic acid, whose ortho- and meta-dihydroxyl groups facilitate efficient electron donation and resonance-stabilized phenoxy radical formation.

### 3.7. In Vitro Cytotoxicity Assessment

**Figure 7:**
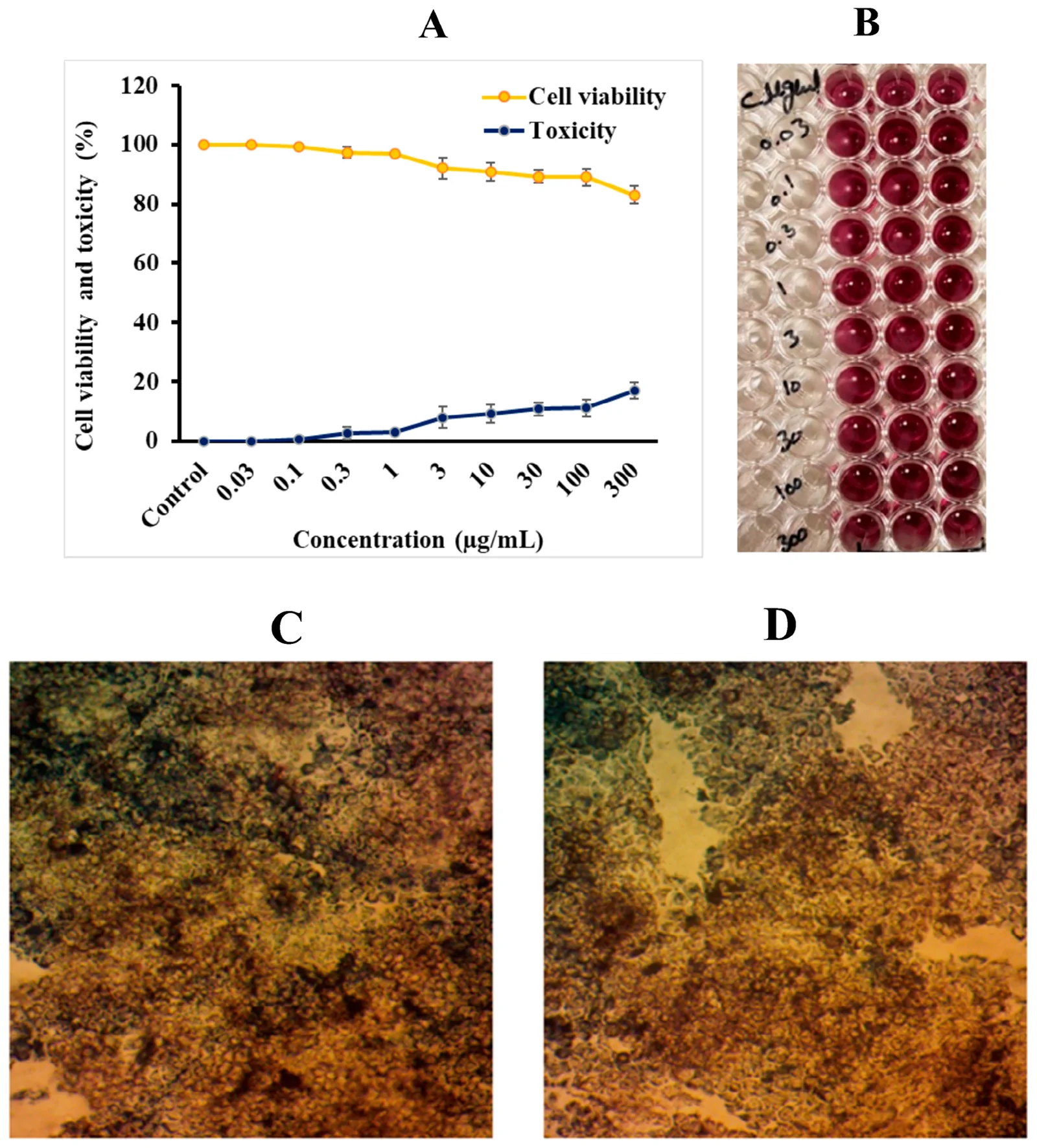
Cytotoxicity assessment of the optimized thyme–moringa–echinacea ternary mixture against OEC cells. (**A**) Percentages of cell viability and toxicity. (**B**) Microtiter plate showing MTT assay across all concentrations, indicative of preserved cell metabolic activity. (**C**,**D**) Microscopic images of OEC cells illustrating normal cell morphology at lower concentrations (**C**). Mild morphological changes at 300 µg/mL (**D**). Data are expressed as mean ± standard deviation.

The optimized blend was evaluated against OEC cells across a nine-point concentration range (0.03–300 µg/mL), revealing a mild, concentration-dependent reduction in viability from 99.98% to 83.12% (Figure 7). Cell viability exceeded 88% across the entire sub-100 µg/mL range, and the CC_50_ could not be determined within the tested range (CC_50_ > 300 µg/mL). Across the entire tested range, cell viability remained at or above 70%, meeting the established threshold for non-cytotoxicity. Light microscopy findings supported these quantitative results. At lower concentrations, cells retained their typical epithelial morphology. Even at 300 µg/mL, only slight increases in granularity and minor changes in cell density were observed, with no clear signs of cytopathic effects.

## 4. Discussion

This study demonstrates that a ternary combination of *T. vulgaris*, *M. oleifera*, and *E. purpurea* ethanolic extracts (Run 14; A = 0.331, B = 0.318, C = 0.352) exerts synergistic, multi-target antimicrobial, antibiofilm, and antioxidant activity while retaining a favorable cytotoxicity profile. Rather than restating the individual outcomes reported in Section 3, the following discussion integrates the phytochemical, statistical, microbiological, and toxicological findings into a coherent mechanistic and translational framework and situates them within the broader context of plant-derived antimicrobial and antibiofilm research.

The HPLC-based phenolic and flavonoid fingerprints obtained for the three species provide the chemical rationale for the synergistic behavior subsequently observed in the mixture design and checkerboard experiments. Each extract contributed a chemically distinct, partially non-overlapping set of bioactive constituents—apigenin-dominant *M. oleifera*, chlorogenic-acid- and ferulic-acid-rich *E. purpurea*, and resorcinol/pyrocatechol-dominant *T. vulgaris*—consistent with the established phytochemical signatures of moringa and echinacea preparations [15]. This compositional complementarity, rather than any single dominant compound, is the most plausible explanation for why the ternary blend consistently outperformed individual extracts and most binary pairs across every bioassay. The conservation of quercetin across all three matrices further suggests a convergent, taxonomically independent antioxidant strategy [14], while the 4-fold enrichment of ferulic acid in *E. purpurea* relative to *T. vulgaris* illustrates how the Lamiaceae-characteristic accumulation of hydroxycinnamic acid diversifies, rather than duplicates, the pooled phytochemical arsenal available to the mixture. While HPLC profiling identified major constituents, it does not establish which compound(s) drive the observed synergy; attribution to specific phenolics/flavonoids is based on precedents in the literature, and bioactivity-guided fractionation would be needed to confirm individual contributions.

A central finding of this work is the convergence of two independently derived optimization approaches—the response-surface mixture design and the checkerboard FIC assay—on the same balanced ternary ratio at the point of maximal antimicrobial efficacy. This concordance is mechanistically meaningful and indicates that the synergy captured statistically by the significant AB, AC, BC, and (for *C. albicans*) ABC interaction terms reflects a real pharmacodynamic phenomenon rather than an artifact of the modeling procedure, in line with recommendations that mixture-design optima be corroborated by an independent interaction metric [24,25,32]. The dominance of the thyme–moringa (AB) interaction in both models is consistent with complementary membrane targeting by thymol/carvacrol-associated compounds and moringa isothiocyanates, which act on distinct but cooperative sites of the microbial envelope [33]. Equally informative is the divergence between the two test organisms: the significant ternary (ABC) term and the negative high-order coefficient for *C. albicans* point to a three-way cooperative effect that plateaus (and even mildly reverses) at very high simultaneous proportions of all three extracts, whereas the *S. mutans* response was adequately captured by a lower-order, purely pairwise model. This organism-specific difference in interaction order suggests that the fungal membrane presents multiple, simultaneously exploitable targets for the three phytochemical classes, while the bacterial target repertoire is more effectively saturated by pairwise combinations alone [34].

The FIC data extend this interpretation. Binary combinations against *C. albicans* ranged from additive to synergistic, whereas two of three binary pairs against *S. mutans* were indifferent, only becoming synergistic once all three extracts were combined. The synergy of the ternary blend is best explained as a sequential, multi-target interaction rather than a simple additive effect. The lipophilic phenolic monoterpenes of *T. vulgaris* (thymol, carvacrol) act first at the envelope, impairing ergosterol biosynthesis and membrane integrity in *C. albicans* and perturbing bilayer packing and permeability in *S. mutans* [35,36,37]. We suggest that this compromised envelope lowers the diffusion barrier for more polar co-actives, such as *M. oleifera*’s flavonoids and glucosinolate-derived isothiocyanates and *E. purpurea*’s caffeic acid derivatives (chiefly cichoric acid), allowing them to reach cytoplasmic targets and suppress adhesion and matrix assembly at concentrations that are sub-inhibitory alone [20]. At the biofilm level, the blend is therefore expected to couple bactericidal/fungicidal envelope damage with anti-virulence action, namely, by interfering with the glucosyltransferase-mediated insoluble glucan synthesis that underpins sucrose-dependent *S. mutans* colonization and suppressing the yeast-to-hypha transition that structurally scaffolds *C. albicans* biofilms [38].

The differential susceptibility observed across *S. mutans* and *C. albicans* is best explained by differences in envelope architecture, rather than by any single potency ranking of the mixture itself. The absence of an outer membrane in Gram-positive *S. mutans* allows direct access of flavonoids such as apigenin, quercetin, and hesperidin to the cytoplasmic membrane, an interpretation consistent with established structure–activity relationships for membrane-active plant phenolics [39,40,41]. The ternary blend’s comparatively reduced, though still appreciable, activity against Gram-negative species reflects the well-documented permeability barrier imposed by the lipopolysaccharide-rich outer membrane, and the particularly high resistance of *P. aeruginosa* is consistent with its constitutive efflux capacity and intrinsic biofilm-forming phenotype, both of which are known to reduce susceptibility to phytochemical agents [41,42,43,44]. That the strongest inhibition across the entire panel was recorded against *C. albicans* is noteworthy—fungal membranes are uniquely enriched in ergosterol, a sterol whose structural features favor intercalation by apigenin and kaempferol far more readily than the cholesterol-free bacterial bilayer, offering a coherent explanation for why the same extract mixture, without any fungal-specific formulation, achieved its greatest effect against a eukaryotic pathogen [35,36,37]. Collectively, this pattern indicates that the ternary mixture acts through a broadly conserved, membrane- and thiol-enzyme-directed mechanism whose apparent potency is modulated, rather than determined, by envelope permeability.

**Figure 8:**
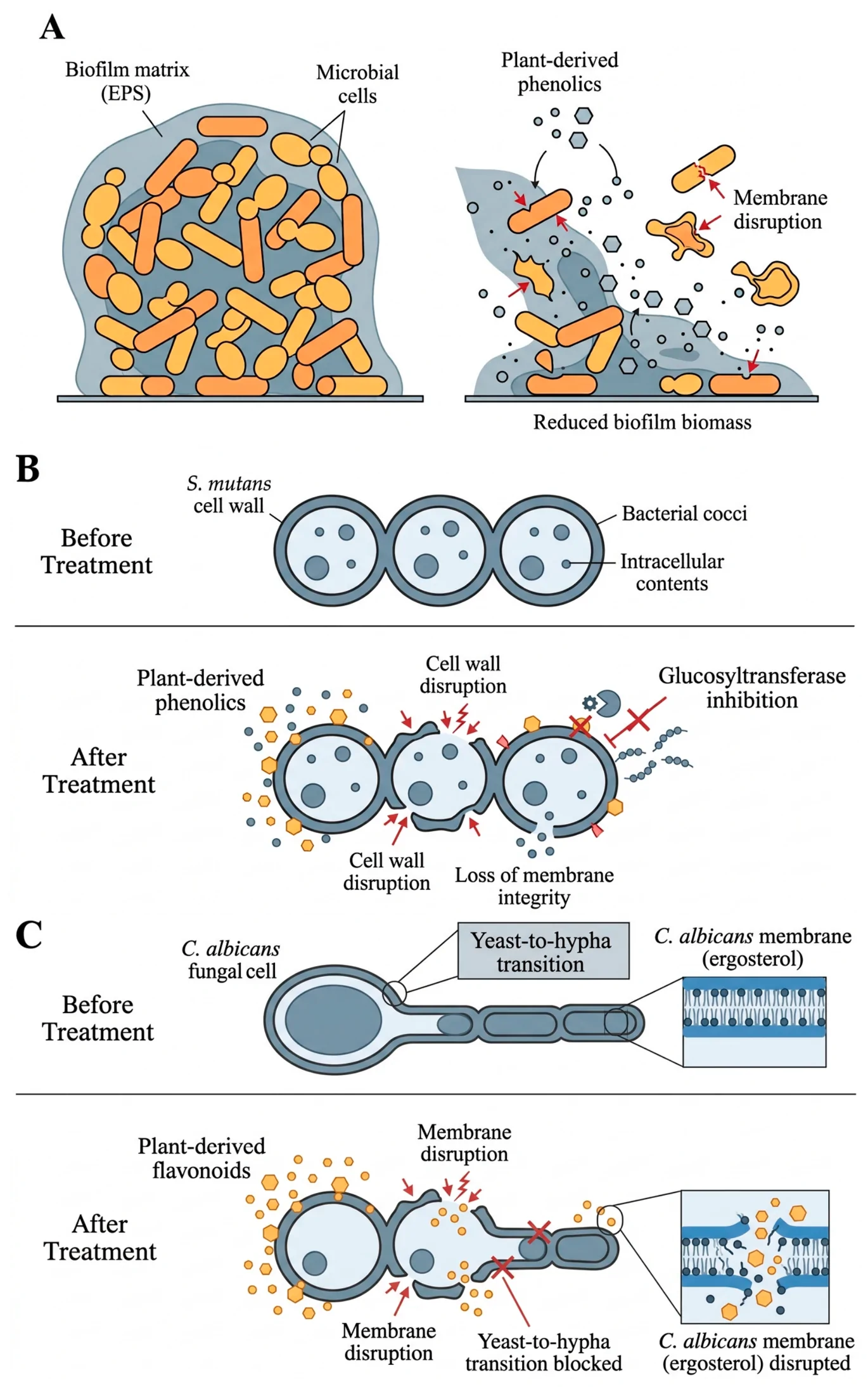

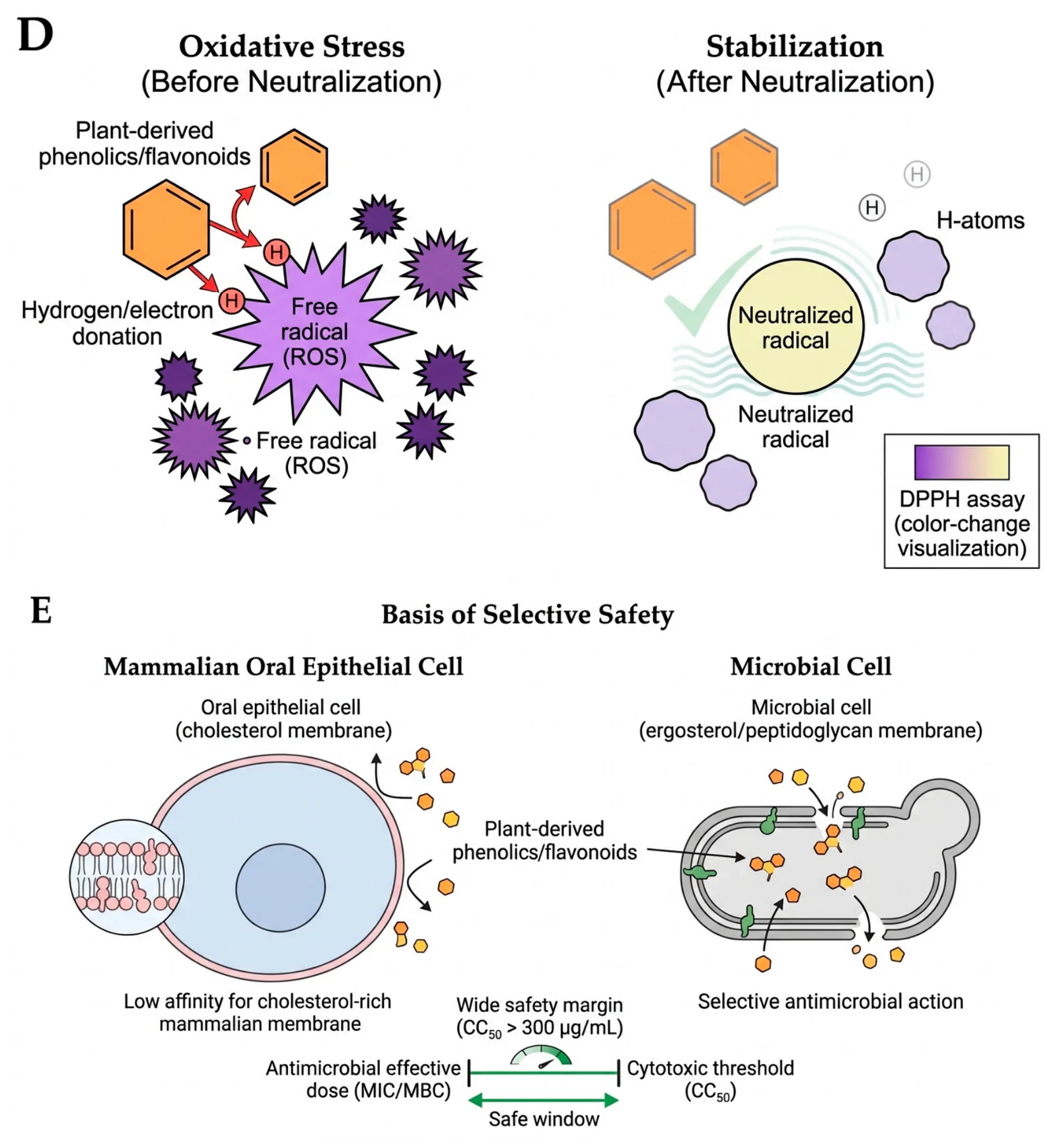
Proposed mechanisms underlying the observed bioactivities of the optimized *T. vulgaris*–*M. oleifera*–*E. purpurea* blend. (**A**) Biofilm disruption. (**B**) Antibacterial mechanism against *S. mutans*, involving cell wall disruption and glucosyltransferase inhibition. (**C**) Antifungal mechanism against *C. albicans*, involving ergosterol-directed membrane disruption and suppression of the yeast-to-hypha transition. (**D**) Antioxidant radical-scavenging mechanism. (**E**) Proposed basis for selective mammalian cell safety, based on differential membrane composition.

Considered together, the antibiofilm and time–kill datasets reveal that the ternary mixture’s activity is not confined to planktonic growth inhibition but extends to both the structural and kinetic dimensions of infection control. The near-complete biofilm inhibition achieved against *C. albicans* at the MIC substantially exceeds the 60–75% range typically reported for single-plant phenolic extracts, supporting the interpretation that the multi-extract formulation confers antibiofilm activity beyond what any individual component would be expected to achieve [45,46]. Based on prior reports, this may involve combined suppression of the Efg1/Cph1-regulated yeast-to-hypha transition by apigenin and quercetin together with ergosterol-directed membrane intercalation by chlorogenic and ferulic acids. These two complementary routes are consistent with our findings, though they are not directly demonstrated to be potential contributors to the observed reduction in Candida biofilm formation [47,48,49,50,51,52]. The proposed mechanisms underlying these bioactivities, synthesized from the identified phytochemical profile and supporting literature, are summarized schematically in Figure 8.

The time–kill kinetics reinforce and extend this biofilm picture. The biphasic killing profile against *C. albicans*—that is, rapid elimination of the bulk population within the first six hours followed by slower clearance of a residual, more tolerant subpopulation—is characteristic of a primary membrane-lytic mechanism acting on a heterogeneous fungal population and is consistent with the greater susceptibility of ergosterol-rich membranes to phenolic intercalation relative to bacterial lipid bilayers [53,54,55]. In contrast, the slower, near-linear bactericidal kinetics observed against *S. mutans* suggest a mechanistically distinct, more gradual mode of action; based on the literature, this pattern is consistent with glucosyltransferase inhibition and quorum-sensing attenuation, rather than rapid membrane lysis, although these mechanisms were not directly tested here [4,56]. This organism-specific divergence in killing kinetics—fungicidal and rapid for *C. albicans*, bactericidal but delayed for *S. mutans*—mirrors the divergence already noted in the mixture design interaction structure and the FIC classification. Together, these three independent lines of evidence converge on a consistent picture: the ternary extract engages fungal and bacterial targets through fundamentally different rate-limiting mechanisms, even though both are ultimately neutralized at the optimized ternary ratio. From an applied standpoint, the co-occurrence of *C. albicans* and *S. mutans* in mixed oral biofilms associated with dental caries and oral candidiasis lends particular relevance to a formulation capable of concentration-dependent control over both organisms simultaneously.

The DPPH radical-scavenging ranking (moringa > echinacea > thyme) tracked closely with the relative abundance of ortho- and meta-dihydroxylated phenolics, particularly pyrocatechol, resorcinol, and chlorogenic acid, across the three extracts and is consistent with prior attribution of *M. oleifera*’s antioxidant capacity to apigenin- and chlorogenic-acid-type constituents and of *E. purpurea*’s antioxidant activity to caffeic acid derivatives such as chlorogenic and caftaric acid [15,57]. The comparatively lower antioxidant activity of *T. vulgaris*, despite its distinct ferulic-acid-driven antimicrobial contribution, illustrates that antioxidant and antimicrobial potency are not simply co-linear properties of a single dominant compound but instead arise from partially separable phytochemical subsets within the same extract [58]. Because oxidative stress and microbial colonization frequently co-occur at infected or inflamed epithelial surfaces, the retention of substantial radical-scavenging capacity alongside potent antimicrobial and antibiofilm activity is a functionally relevant, rather than incidental, property of the optimized ternary mixture and supports its consideration for applications where both oxidative and microbial burden require concurrent management, such as oral or dermal care (Figure 8).

A key requirement for any natural antimicrobial candidate is that inhibitory concentrations be achievable well below those causing host cell toxicity. In this respect, the wide margin between the sub-MIC and MIC concentrations effective against *C. albicans* and *S. mutans* (in the low micrograms-per-milliliter to sub-milligram range) and the concentrations required to produce any appreciable reduction in OEC cell viability (CC50 > 300 µg/mL, viability ≥ 88% across the entire sub-100 µg/mL range) is a strong indicator of selective action toward microbial rather than mammalian targets. This safety margin is consistent with the low intrinsic cytotoxicity widely reported for the phenolic constituents identified by HPLC, including apigenin, quercetin, chlorogenic acid, ferulic acid, and vanillic acid, at concentrations well above their antimicrobial thresholds [59] and with previous cytotoxicity evaluations of polyphenol-rich thyme and moringa preparations [24] (Figure 8). The absence of alkaloids or cytotoxic terpenoids in the phytochemical profile, together with the preserved epithelial morphology observed by light microscopy even at the highest tested concentration, further supports a favorable therapeutic window [60]. This selectivity should nonetheless be interpreted as preliminary; it is based on a single mammalian cell line under short-term in vitro exposure and does not establish safety under repeated or prolonged exposure in more physiologically representative cell systems or in vivo.

## 5. Conclusions

This study demonstrated that mixture design optimization enables the rational identification of a synergistic ternary thyme–moringa–echinacea blend with dual antimicrobial, antibiofilm, and antioxidant activity against two clinically important oral pathogens at concentrations that are well tolerated by mammalian cells in vitro. These results support this blend’s potential as a candidate for further development into plant-based oral care or antimicrobial formulations, offering a multi-target alternative to single-extract approaches amid rising antimicrobial resistance. Translation will require validation against clinical isolates, in vivo efficacy and safety testing, and mechanistic confirmation of the synergy observed here. This study was limited by its in vitro, reference-strain design; reliance on literature-inferred mechanisms; single-cell-line cytotoxicity testing; use of inhibition zone diameter rather than MIC for optimization; and antibiofilm assessment via crystal violet/SEM without viable cell counting or Live/Dead staining. Future work should validate the blend against resistant clinical isolates; clarify the molecular basis of its ternary synergy through bioactivity-guided fractionation, LC–MS/MS, docking, transcriptomics, proteomics, and enzymatic assays; and conduct further genotoxicity, hemolysis, and organ-specific safety testing.

## Figures and Tables

**Table 1:** L-optimal mixture design matrix.

Run	*T. vulgaris* (A)	*M. oleifera* (B)	*E. purpurea* (C)
1	0.540	0.272	0.188
2	0.521	0.479	0.000
3	0.100	0.660	0.240
4	0.000	0.365	0.635
5	0.117	0.471	0.412
6	0.456	0.099	0.444
7	0.521	0.479	0.000
8	0.660	0.055	0.285
9	0.222	0.214	0.565
10	0.300	0.660	0.040
11	0.340	0.000	0.660
12	0.000	0.365	0.635
13	0.100	0.660	0.240
14	0.331	0.318	0.352
15	0.660	0.055	0.285
16	0.331	0.318	0.352

**Table 2:** L-optimal mixture design runs with actual and predicted inhibition zone diameters (IZD, cm) for *C. albicans* and *S. mutans*.

Run	Mixture Proportions			IZD (cm) *C. albicans*		IZD (cm) *S. mutans*	
	Thyme (A)	Moringa (B)	Echinacea (C)	Actual	Predicted	Actual	Predicted
1	0.540	0.272	0.188	2.70	2.72	3.70	3.69
2	0.521	0.479	0.000	2.50	2.55	3.40	3.39
3	0.100	0.660	0.240	1.70	1.71	2.60	2.71
4	0.000	0.365	0.635	1.20	1.20	2.40	2.30
5	0.117	0.471	0.412	2.50	2.47	3.60	3.42
6	0.456	0.099	0.444	2.30	2.30	3.50	3.12
7	0.521	0.479	0.000	2.60	2.55	3.30	3.39
8	0.660	0.055	0.285	1.50	1.49	2.40	2.29
9	0.222	0.214	0.565	2.10	2.08	3.10	3.25
10	0.300	0.660	0.040	2.00	1.98	3.20	2.99
11	0.340	0.000	0.660	0.90	0.91	1.80	1.91
12	0.000	0.365	0.635	1.20	1.20	2.30	2.30
13	0.100	0.660	0.240	1.70	1.71	2.60	2.71
14	0.331	0.318	0.352	3.10	3.06	4.10	4.06
15	0.660	0.055	0.285	1.50	1.49	2.10	2.29
16	0.331	0.318	0.352	3.00	3.06	3.80	4.06

**Table 3:** ANOVA and model fit statistics for the special cubic (*C. albicans*) and quadratic (*S. mutans*) mixture response models.

Source	*C. albicans* (Special Cubic)					*S. mutans* (Quadratic)				
	SS	df	MS	F	*p*-Value	SS	df	MS	F	*p*-Value
Model	6.80	6	1.13	776.69	<0.0001 *	6.61	5	1.32	31.92	<0.0001 *
Linear mixture	1.62	2	0.81	554.57	<0.0001 *	1.10	2	0.55	13.21	0.0016 *
AB	0.769	1	0.769	526.70	<0.0001 *	4.88	1	4.88	117.78	<0.0001 *
AC	0.331	1	0.331	226.63	<0.0001 *	2.71	1	2.71	65.37	<0.0001 *
BC	0.448	1	0.448	307.26	<0.0001 *	3.30	1	3.30	79.72	<0.0001 *
ABC	0.0523	1	0.0523	35.82	0.0002 *	–	–	–	–	–
Residual	0.0131	9	0.0015	–	–	0.4145	10	0.0415	–	–
Lack of Fit	0.0031	4	0.0008	0.39	0.8075 ns	0.3145	5	0.0629	3.15	0.1171 ns
Pure Error	0.0100	5	0.0020	–	–	0.1000	5	0.0200	–	–
Cor Total	6.81	15	–	–	–	7.03	15	–	–	–

* Significant (*p* < 0.05); ns: not significant. SS = sum of squares; df = degrees of freedom; MS = mean square; F = F-value. Fit statistics: *C. albicans*, R^2^ 0.9981, Adj. R^2^ 0.9968, Pred. R^2^ 0.9944, Adeq. Precision 84.823, CV% 1.88, Mean 2.03 cm, Std. Dev. 0.0382; *S. mutans*, R^2^ 0.9410, Adj. R^2^ 0.9115, Pred. R^2^ 0.8410, Adeq. Precision 17.197, CV% 6.80, Mean 2.99 cm, Std. Dev. 0.2036.

**Table 4:** Fractional inhibitory concentration (FIC) indices for binary and ternary combinations of thyme, moringa, and echinacea extracts against *C. albicans* and *S. mutans*.

Combination	Single MIC (mg/mL)	Combined MIC (mg/mL)	Individual FIC	FICI	Interaction
**Against *Candida albicans* (Antifungal)**					
A/B	0.125/0.125	0.0625/0.0625	0.50/0.50	1.00	Additive
A/C	0.125/0.500	0.03125/0.125	0.25/0.25	0.50	Synergy
B/C	0.125/0.500	0.03125/0.250	0.25/0.50	0.75	Additive
A/B/C	0.125/0.125/0.500	0.015625/0.03125/0.0625	0.125/0.25/0.125	0.50	Synergy
**Against *Streptococcus mutans* (Antibacterial)**					
A/B	0.125/0.125	0.125/0.0625	1.00/0.50	1.50	Indifferent
A/C	0.125/0.500	0.0625/0.250	0.50/0.50	1.00	Additive
B/C	0.125/0.500	0.125/0.250	1.00/0.50	1.50	Indifferent
A/B/C	0.125/0.125/0.500	0.03125/0.015625/0.0625	0.25/0.125/0.125	0.50	Synergy

Fractional inhibitory concentration (FIC) indices for binary and ternary combinations of thyme (A), moringa (B), and echinacea (C) extracts against *C. albicans* and *S. mutans*. All combined MIC values fall on 2-fold serial-dilution steps consistent with standard checkerboard broth microdilution. FICI classification: ≤0.5 = synergy; >0.5–1.0 = additive; >1–4 = indifferent; >4 = antagonism.

## Data Availability

The original contributions presented in this study are included in the article/Supplementary Material. Further inquiries can be directed to the corresponding author.

## Supplementary Materials

The following supporting information can be downloaded at: https://www.mdpi.com/article/10.3390/microorganisms14092010/s1, Figure S1. HPLC detection and quantification of extracts: Phenolic content (A) and Flavonoid content (B) of *Thymus vulgaris*; Phenolic content (C) and Flavonoid content (D) of *Moringa oleifera*; Phenolic content (E) and Flavonoid content (F) of *Echinacea purpurea*.
