# Supplementary material for: Synergistic Antimicrobial and Antibiofilm Activity Optimization of a Thymus vulgaris–Moringa oleifera–Echinacea purpurea Ternary Ethanolic Extract Blend Against Candida albicans and Streptococcus mutans Using L-Optimal Mixture Design
Source: Microorganisms. 2026 Sep 10;14(9):2010. doi: 10.3390/microorganisms14092010 (PMC13609620; doi:10.3390/microorganisms14092010)
Supplement: Supplementary file 1 [file microorganisms-14-02010-s001.zip › microorganisms-4487542-supplementary.pdf]

A

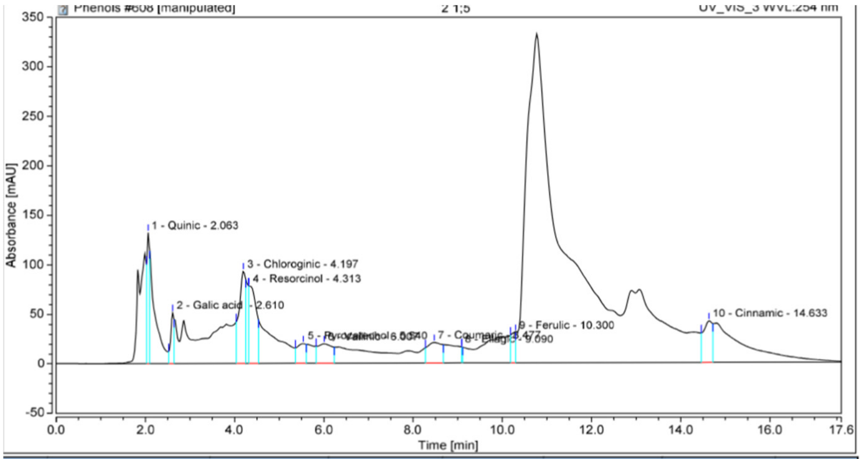

Integration Results

No.	Peak Name	Retention Time min	Area mAU*min	Height mAU	Relative Area %	Relative Height %	Amount ppm
1	Quinic	2.063	7.974	132.643	10.51	26.11	85.19
2	Galic acid	2.610	4.023	51.681	5.30	10.17	31.45
3	Chloroginic	4.197	15.193	93.569	20.03	18.42	232.14
4	Resorcinol	4.313	14.951	78.700	19.71	15.49	298.54
5	Pyrocatechol	5.540	4.658	20.218	6.14	3.98	248.28
6	Vailinic	6.007	7.555	20.091	9.96	3.95	21.09
7	Coumaric	8.477	7.813	21.136	10.30	4.16	14.21
8	Ellagic	9.090	0.383	16.537	0.50	3.25	0.51
9	Ferulic	10.300	3.530	31.132	4.65	6.13	14.58
10	Cinnamic	14.633	9.772	42.355	12.88	8.34	34.81
**Total:**			**75.852**	**508.063**	**100.00**	**100.00**	

B

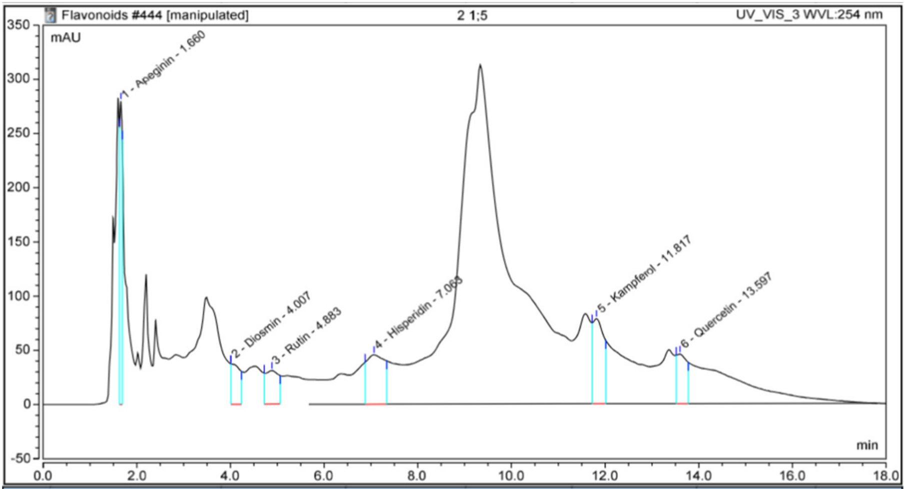

Integration Results

No.	Peak Name	Retention Time min	Area mAU*min	Height mAU	Relative Area %	Relative Height %	Amount ppm
1	Apeginin	1.660	16.071	279.938	18.81	54.05	321.0485
2	Diosmin	4.007	7.913	37.504	9.26	7.24	0.2472
3	Rutin	4.883	9.956	30.815	11.65	5.95	2.5781
4	Hisperidin	7.063	19.680	45.524	23.03	8.79	17.4083
5	Kampferol	11.817	20.740	78.552	24.28	15.17	6.8933
6	Quercetin	13.597	11.076	45.595	12.96	8.80	13.9273
**Total:**			**85.437**	**517.929**	**100.00**	**100.00**	

C

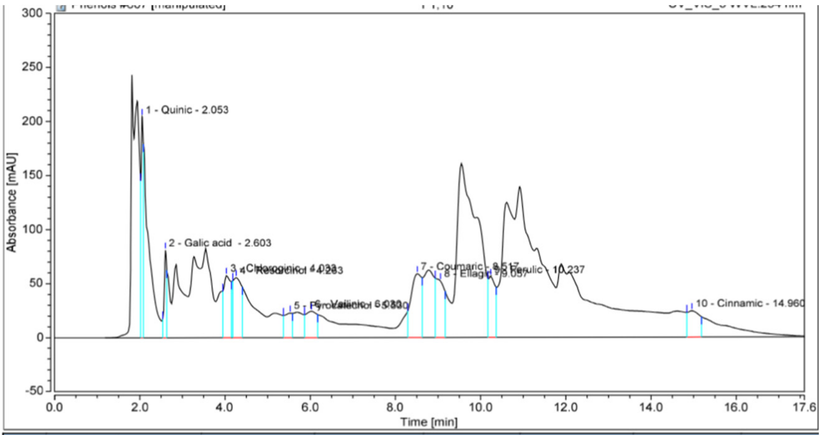

Integration Results							
No.	Peak Name	Retention Time min	Area mAU*min	Height mAU	Relative Area %	Relative Height %	Amount ppm
1	Quinic	2.053	11.573	204.549	11.94	32.11	123.62
2	Galic acid	2.603	4.904	81.064	5.06	12.72	38.33
3	ChlorogInic	4.033	10.570	57.487	10.91	9.02	161.51
4	Resorcinol	4.263	11.797	55.432	12.17	8.70	235.57
5	Pyrocatechol	5.530	4.661	22.641	4.81	3.55	248.46
6	Vailinic	6.030	7.044	24.359	7.27	3.82	19.66
7	Coumaric	8.517	16.587	58.867	17.12	9.24	30.17
8	Ellagic	9.057	11.704	52.150	12.08	8.19	15.52
9	Ferulic	10.237	10.342	56.229	10.67	8.83	42.72
10	Cinnamic	14.960	7.728	24.338	7.97	3.82	27.53
**Total:**			**96.911**	**637.114**	**100.00**	**100.00**	

D

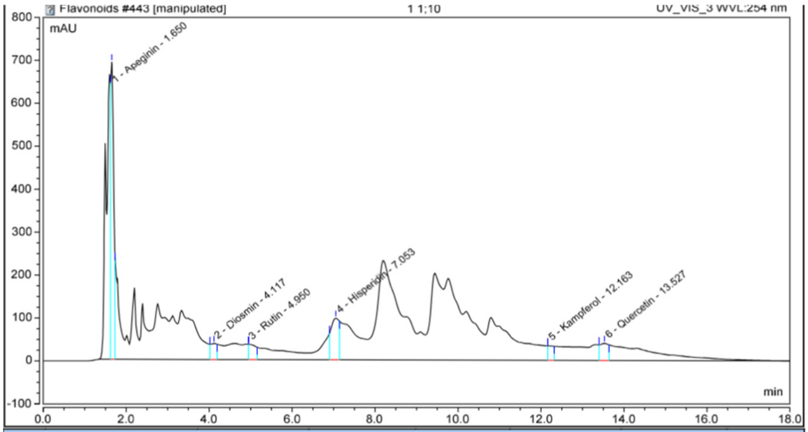

Integration Results							
No.	Peak Name	Retention Time min	Area mAU*min	Height mAU	Relative Area %	Relative Height %	Amount ppm
1	Apeginin	1.650	57.759	692.843	54.29	74.03	1153.8176
2	Diosmin	4.117	6.195	37.259	5.82	3.98	0.1935
3	Rutin	4.950	6.848	35.649	6.44	3.81	1.7732
4	Hisperidin	7.053	21.219	96.857	19.94	10.35	18.7696
5	Kampferol	12.163	5.210	33.493	4.90	3.58	1.7318
6	Quercetin	13.527	9.156	39.770	8.61	4.25	11.5122
**Total:**			**106.388**	**935.869**	**100.00**	**100.00**	

E

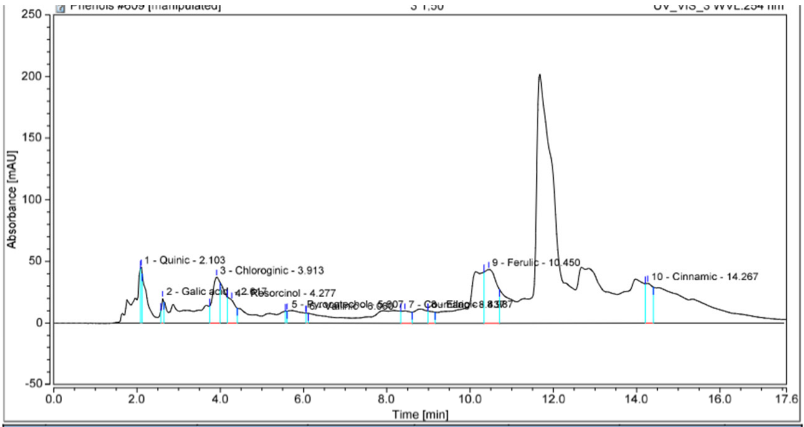

Integration Results							
No.	Peak Name	Retention Time min	Area mAU*min	Height mAU	Relative Area %	Relative Height %	Amount ppm
1	Quinic	2.103	1.615	45.424	4.06	19.31	17.25
2	Galic acid	2.617	1.190	20.093	2.99	8.54	9.30
3	Chloroginic	3.913	7.244	37.389	18.20	15.89	110.69
4	Resorcinol	4.277	4.111	18.693	10.33	7.95	82.10
5	Pyrocatechol	5.607	0.423	9.814	1.06	4.17	22.53
6	Vailinic	6.060	0.428	8.153	1.07	3.47	1.19
7	Coumaric	8.437	2.632	9.896	6.61	4.21	4.79
8	Ellagic	8.987	1.641	9.874	4.12	4.20	2.18
9	Ferulic	10.450	14.382	43.617	36.12	18.54	59.41
10	Cinnamic	14.267	6.150	32.323	15.45	13.74	21.91
**Total:**			**39.816**	**235.275**	**100.00**	**100.00**	

F

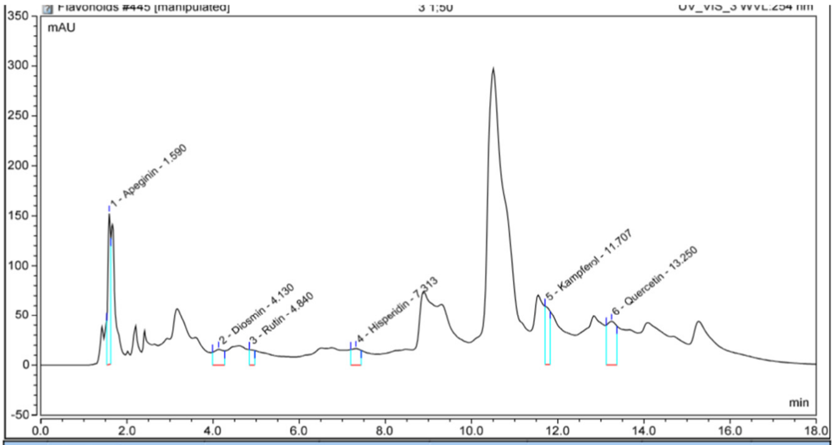

Integration Results							
No.	Peak Name	Retention Time min	Area mAU*min	Height mAU	Relative Area %	Relative Height %	Amount ppm
1	Apeginin	1.590	10.700	151.795	28.35	50.32	213.7393
2	Diosmin	4.130	4.133	15.670	10.95	5.19	0.1291
3	Rutin	4.840	1.919	15.470	5.08	5.13	0.4968
4	Hisperidin	7.313	3.858	16.429	10.22	5.45	3.4125
5	Kampferol	11.707	6.644	58.638	17.61	19.44	2.2083
6	Quercetin	13.250	10.483	43.650	27.78	14.47	13.1809
**Total:**			**37.736**	**301.652**	**100.00**	**100.00**	

Figure S1. HPLC detection and quantification of extracts: Phenolic content (A) and Flavonoid content (B) of *Thymus vulgaris*; Phenolic content (C) and Flavonoid content (D) of *Moringa oleifera*; Phenolic content (E) and Flavonoid content (F) of *Echinacea purpurea*
